# 

**Published:** 1901-03-02

**Authors:** 


### NOTICE TO CORRESPONDENTS.

All MSS., Letters, Books for Review, and other matters intended for the Editor should be addressed The Editor, "The Hospital," The Hospital Building, 28 & 29 Southampton Street, Strand, W.C.

The Editor cannot undertake to return rejected MS., even when accompanied by stamped directed envelope.

**Notice to Advertisers and Subscribers.**

All Advertisements, Orders for Copies of The Hospital, and Business Communications should be addressed to **THE MANAGER** (**Not to the Editor**), The Hospital, 28 & 29 Southampton Street Strand, London, W.C.

The Cost of Subscription, post free, is as follows:—Per week, 2½d.; per quarter, 2s. 8d.; per half-year, 5s. 3d.; per annum, 10s. 6d. Foreign:—Per annum, 15s. 2d., payable, in all cases, in advance.

**NOTICE.—Vol. XXVIII. of "The Hospital" (April to September, 1900), in handsome cloth binding, is now on sale at the Office of this Journal, price, 7s. 6d. Cases for binding the Numbers contained in this Volume 1s. 6d. Index to Vol. XXVIII., 2½d., post free.**

**The Monthly Part for March is now ready. Price 6d., by post 8d.**

### BOOKS RECEIVED.

Review of Reviews Office.

"Stories of the Queen: An Anecdotal History of the Reign."

The Scientific Press, Limited.

"The Nursing Profession: How and Where to Train." By Sir Henry Burdett, K.C.B.

British Medical Association.

"Report of the Anæsthetic Commission."

Baillière, Tindall, and Cox.

"Arsenical Poisoning in Beer Drinkers." By T. N. Kelynack, M.D., M.R.C.P., and William Kirby, F.L.S.

"Practical Guide to the Public Health Acts." By Thos. Whiteside Hime, B.A., M.D. Second much enlarged edition.

"Mock Nurses of the Latest Fashion, A.D. 1901." By Frederick James Gant, F.R.C.S. Second edition.

"Physical Diagnosis of Diseases of the Chest." By Richard C. Cabot, M.D.

Gory and Bird.

"The No-Breakfast Plan."

"Chronic Alcoholism." By G. H. Dewey, M.D.

### Scraps and Gleanings.

The second interim report of the Arbroath District Committee of the County Council in connection with the proposed erection of a fit epidemic hospital for the Burgh of Arbroath and the Arbroath District has just been handed in. An attempt was made to reduce the cost of the hospital by reducing the size, but the Local Government Board declined to sanction any such reduction, and suggested instead some small economies in connection with the administrative block. As, however, these economies would only reduce the cost by £160, it was decided to revert to the original plan, the gross cost of which, including furnishing, would be £12,200 odd.

### The Student of Medicine.

### APPOINTMENTS.

**Francis J. Allen,** M.D., D.P.H., F.R.S.E., appointed Medical Officer of Health for the City of Westminster. **E. W. Allsom,** L.R.C.P., L.R.C.S.Edin., appointed Extern Physician to the South Charitable Infirmary and County Hospital, Cork. **F. S. Beachcroft,** M.R.C.S., L.R.C.P.Lond., appointed Certifying Factory Surgeon for the Petworth District of Sussex. **A. E. Berry,** M.D., appointed Medical Officer of Health for the Barton-upon-Irwell Rural District. **W. A. Bond,** M.A., M.D.Camb., D.P.H., appointed Medical Officer of Health to the Holborn Borough Council. **George Bower,** L.R.C.P., L.R.C.S.Edin., D.P.H.Vict., ex-M.O.H. Borough of Macclesfield, appointed Medical Officer of Health, Sutton Urban Sanitary District, Surrey. **R. C. Brown,** M.B., B.C.Camb., appointed Medical Officer for the Fifth District of the Depwade Union. **R. K. Brown,** B.A., M.B., B.Ch.R.U.I., appointed Medical Officer of Health for the Borough of Bermondsey. **Warburton Brown,** L.R.C.P., M.R.C.S., L.D.S., appointed Junior Dental Surgeon to the Seamen's Hospital Society, Greenwich, S.E. **Lesli[illegible] Buchanan,** M.D., appointed Surgeon to the Glasgow Ey[illegible] Infirmary. **P. J. Cammidge,** M.R.C.S.Eng., L.R.C.P.Lond[illegible] appointed Bacteriologist on the Staff of the County Medic[illegible] Officer for the West Riding of Yorkshire. **Baker Lyste[illegible]**

**Cole**, B.A.Dub., M.D., B.Ch., appointed Honorary Assistant Physician to the Royal Portsmouth Hospital. **M. W. Compton**, M.R.C.S., L.R.C.P.Lond., appointed Certifying Factory Surgeon for the Keyingham District of the East Riding of York. **Wellesley Coombs**, F.R.C.S.E., appointed Honorary Anæsthetist to the Worcester General Infirmary. **J. H. Crocker**, M.D.Vict., appointed Medical Officer of Health for the Borough of Richmond, Surrey. **W. L. Bowen Davis**, M.R.C.S., L.R.C.P.Lond., appointed Honorary Medical Officer to the Denbighshire Infirmary. **A. Dingwall**, M.A., M.B., C.M.Aberd., appointed Certifying Factory Surgeon for the Presteigne District, co. Radnor. **H. B. Dismorr**, M.R.C.S.Eng., L.R.C.P.Lond., appointed Senior Assistant Medical Officer at the Lewisham Infirmary. **Oliver Eaton**, M.R.C.S., L.R.C.P.Lond., D.P.H.Camb., appointed Certifying Factory Surgeon for the Exmouth District of Devonshire. **George Elder**, M.D.Glas., appointed Consulting Surgeon to the Samaritan Hospital for Women, Nottingham. **C. A. Ensor**, M.R.C.S., L.R.C.P.Lond., appointed Certifying Factory Surgeon for the Tisbury District of Wilts. **Rosa Ford**, M.B.Lond., appointed Resident Medical Officer to the York Dispensary. **Kenneth W. Goadby**, M.R.C.S., L.R.C.P., L.D.S.Eng., appointed Senior Dental Surgeon to the Seamen's Hospitals. **Wm. Sampson Handley**, M.S., M.D.Lond., F.R.C.S.Eng., appointed Surgeon to Out-patients at the Samaritan Free Hospital for Women and Children, Marylebone Road. **Leonard Harman**, M.B.&C., appointed District Medical Officer and Public Vaccinator for the Coombe and Ham Districts of the Kingston-on-Thames Union. **J. F. Hodgson**, M.B., Ch.B.Vict., appointed Senior House Surgeon to the Oldham Infirmary. **Katie Welton Hogg**, B.A.Sydney, M.B., Ch.B.Edin., L.M., Coombe Hospital, Dublin, appointed Assistant to the Master, Coombe Hospital, Dublin. **L. A. W. Johnston**, M.R.C.S.Eng., L.R.C.P.Lond., appointed Assistant Medical Officer at the City Fever Hospital, Lodge Road, Birmingham. **W. H. Kelson**, M.D., B.S.Lond., F.R.C.S.Eng., appointed Assistant Surgeon to the London Throat Hospital. **C. C. Lall**, M.B., C.M.Edin., appointed Assistant Resident Medical Officer to the Salford Union Infirmary. **G. J. Lane**, M.D.Dub., appointed Medical Officer for the Heyford District of the Bicester Union. **J. Mackenzie**, L.R.C.P., L.R.C.S.Edin., appointed Medical Officer for the Bentham District of the Settle Union. **J. M. Martin**, M.B., B.C., D.P.H., appointed Medical Officer of Health for the Nailsworth Urban District. **L. H. McGavin**, F.R.C.S.Eng., appointed Assistant Surgeon to the Hospital for Women, Soho Square, W.C. **T. Moore**, M.R.C.S.Eng., appointed Medical Officer of Health for the Hazel Grove and Bramhall Urban District. **J. Foster Palmer**, L.R.C.P.Lond., M.R.C.S.Eng., appointed Public Vaccinator for Chelsea. **G. F. Philpot**, M.R.C S.Eng., appointed Medical Officer for the Belbroughton District of the Bromsgrove Union. **E. O. Price**, M.D.Edin., appointed Certifying Factory Surgeon for the Bangor District of Carnarvonshire. **J. Lloyd Roberts**, M.B., C.M.Edin., appointed Honorary Consulting Medical Officer to the Denbighshire Infirmary. **P. Caldwell Smith**, M.D.Glasg., D.P.H.Camb., appointed Medical Officer of Health for Borough of Wandsworth. **Edward Smyth**, L.R.C.P., L.R.C.S.Edin., appointed Medical Officer for the Dispensary District of Portaferry. **Munro E. Tyrrell**, M.D.Edin., appointed Medical Officer to the Eastlands Infectious Diseases Hospital, Galashiels. **A. S. Whytock**, M.A., M.B., Ch.B.Edin., appointed resident House-Surgeon to the Brighton and Hove Hospital for Women. **L. J. Willan**, M.R.C.S.Eng., appointed District Medical Officer of the Dorking Union. **Thomas Williams**, L.R.C.P., L.R.C..S.Irel., appointed Certifying Factory Surgeon for Masham District of the North Riding of York.

Ealing Cottage Hospital and Dispensary.—The following have been appointed Dental Surgeons: **Edward Carter**, L.D.S., R.C.S.Eng., F.P.S.Glas.; **Walter Green**, L.D.S., R.C.S.Eng.; **Leslie M. Stocken**, L.R.C.P.Lond., M.R.C.S.&L.D.S.Eng.; **Herbert R. Bowtell**, L.R.C.P.Lond., M.R.C.S.&L.D.S.Eng.

